# Coordination on Networks: Does Topology Matter?

**DOI:** 10.1371/journal.pone.0055033

**Published:** 2013-02-06

**Authors:** Alberto Antonioni, Maria Paula Cacault, Rafael Lalive, Marco Tomassini

**Affiliations:** Faculty of Business and Economics, University of Lausanne, Lausanne, Switzerland; Universidad Carlos III de Madrid, Spain

## Abstract

Effective coordination is key to many situations that affect the well-being of two or more humans. Social coordination can be studied in coordination games between individuals located on networks of contacts. We study the behavior of humans in the laboratory when they play the Stag Hunt game – a game that has a risky but socially efficient equilibrium and an inefficient but safe equilibrium. We contrast behavior on a cliquish network to behavior on a random network. The cliquish network is highly clustered and resembles more closely to actual social networks than the random network. In contrast to simulations, we find that human players dynamics do not converge to the efficient outcome more often in the cliquish network than in the random network. Subjects do not use pure myopic best-reply as an individual update rule. Numerical simulations agree with laboratory results once we implement the actual individual updating rule that human subjects use in our laboratory experiments.

## Introduction

Game theory [1] is a useful tool in the study of economic, social, and biological situations for describing interactions between agents having possibly different and often conflicting objectives. Paradigmatic games such as the Prisoner’s Dilemma have been used in order to represent the tension that appears in society when individual objectives are in conflict with socially desirable outcomes. Indeed, most of the vast research literature has focused on conflicting situations in order to uncover the mechanisms that could lead to cooperation instead of socially harmful outcomes. However, there are important situations in social and economic contexts that do not require players to use aggressive strategies but simply to coordinate their actions on a common goal, since in many cases the best course of action is to conform to the standard behavior. For example, if one is used to drive on the right side of the road and travels to a country where the norm is reversed, it pays off to follow the local norm. Games that express this extremely common kind of interactions are called *coordination games*.

One important consideration is the interaction structure of the playing agents. It is frequently assumed, especially in theoretical work but also in many laboratory experiments, that agents interact globally with any other agent in the population. However, everyday observation tells us that in animal and human societies, individuals usually tend to interact more often with some specified subset of partners; for instance, teenagers tend to adopt the fashions of their close friends group; closely connected groups usually follow the same religion, and so on. In short, social interaction is mediated by networks, in which vertices identify people, firms etc., and edges identify some kind of relation between the concerned vertices such as friendship, collaboration, economic exchange and so on. Thus, locality of interaction plays an important role. The dynamical behavior of games on networks has been investigated both theoretically and by numerical simulation methods (see [2]–[6] for comprehensive recent reviews).

Several analytically rigorous results are available for coordination games in well-mixed populations [7], [8], as well as populations with a simple local interaction structure such as rings and grids [9], [10]. These results are very useful and will be summarized later on; however, while game theory has normative value, its prescriptions are not always reflected in the way people act when confronted with these situations. This has been made manifest by a host of results of experiments with people [11]. Coordination games are no exception and also confront the theory with many puzzles. For coordination games on small-worlds and regular networks the laboratory experiments carried out in [12] and in [13]–[15] are particularly relevant.

In this paper we describe and discuss a laboratory experiment on coordination games using particular local network structures that are characteristic of real social interactions and thus go beyond the simple and well-known rings and grids usually employed in such experiments. By doing so we wish to understand how well the theoretical or simulated actions of automata align with choices by humans. If theory and simulations line up well with actual choices in the laboratory this re-inforces the use of these relatively cheap tools to understanding behavior. In contrast, if the laboratory reveals results that are not to be expected from theory or simulations, there is a need to refine theory and simulation methods.

The paper is organized as follows. In the next section we present a brief introduction to the subject of coordination games and we summarize the main known theoretical results in order to provide the right context for the experimental part. The following sections deal with the main theme of the present study where, after a discussion of previous related work, we present the setup and the results of our laboratory experiment related to the relevant network structures. Finally, we present a detailed discussion of the results in the context of related work and give our conclusions.

### Coordination Games

As in most previous work, we shall restrict ourselves to two-person, two-strategies, symmetric coordination games. General two-person, two-strategies coordination games have the normal form of Table 1. Here we shall assume that 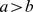 and 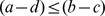; then 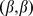 is the risk-dominant equilibrium, while 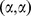 is the Pareto-dominant one. This simply means that players get a higher payoff by coordinating on 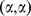 but they risk less by using strategy 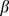 instead. There is also a third equilibrium in mixed strategies but it is evolutionarily unstable. The Pareto-efficient equilibrium 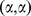 is socially preferable but mis-coordination may happen easily leading to inefficient outcomes. This type of game is the so-called Stag-Hunt game [16]; it has been extensively studied analytically using stochastic processes [7], [9] and by numerical simulation on several model network types [6], [16], [17].

**Table 1:** A general two-person, two-strategies symmetric game.

	*α*	*β*
*α*	*a, a*	*c, d*
*β*	*d, c*	*b, b*

In well-mixed populations, agents may use *myopic best-reply* [8] to revise their strategy. This is a deterministic, bounded-rationality adaptive learning rule in which, in each time step, an agent has the opportunity of revising her current strategy with probability 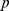. She does so by considering the previous actions of the rest of the population and switches to the action that would maximize her payoff if the other players stick to their previous choices. In other words, 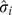 is a myopic best-reply for player 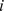 if 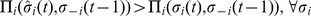, where 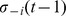 is the strategy profile of all players other than 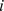 at time 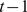. In case of a tie, agent 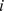 keeps its current strategy.

For best reply both monomorphic populations of all 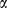 and all 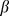 are asymptotically stable states [18]. However, if some noise is introduced in best response dynamics to simulate strategy update errors of various kinds then the stochastically stable state in the long run will be the risk-dominant strategy 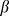 since the risk-dominant strategy has the largest basin [7], [8].

When the population has a network structure the strategy-revision rule described above is slightly modified in such a way that it works for pairs of agents that are neighbors [6], [8], [9]. For populations structured as rings, the risk-dominant strategy 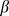 should take over the population in the long run [8], [9] if the agents play according to myopic best-reply. If, instead, agents imitate the strategy of their most successful neighbor and the neighborhood size is large enough, then the payoff-dominant strategy becomes the unique long-run equilibrium [4]. In two-dimensional grids, both equilibria can be reached depending on the evolution rules considered and, most remarkably, dimorphic states, i.e. population states in which 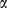 and 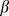 players coexist in a stable manner, become possible [4], [10], [16], [19]. An important feature of these local models is that the convergence is faster than in global interaction models [4].

No general theoretical results on coordination games are available for arbitrary networks. However, the simulation results show that the presence of a local interaction structure provided by a network tends to increase the region of the game’s parameter space in which the Pareto-dominant outcome prevails [6], [16]. Moreover, dimorphic populations may be stable in complex networks thanks to the existence of recognizable communities of tightly linked agents [20].

The conclusion of this brief summary on theoretical results is that either the all-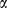 or all-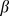 convention can be reached as a stable state in well-mixed populations depending on details such as agent matching, noise, and strategy revision rule. On rings, the stable state in the long-run is most probably the risk-efficient equilibrium all-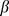, although all-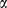 can also arise if agents imitate the best neighbor and neighborhoods are large. On grids and complex networks in general both monomorphic and dimorphic populations can be stable, thus both strategies can coexist.

### Previous Experimental Results on Coordination Games with Local Interactions

We have seen that theory alone is not discriminating enough to solve the equilibrium selection problem by analytical means and thus empirical approaches are very valuable. Indeed, coordination games have been the object of a number of experimental works in the last two decades. Among the most well-known studies dealing with randomly mixing populations and groups, we may cite e.g. [21]–[25] and chapter seven of Camerer’s book [11], where an informative summary is provided.

Given the focus of our work, we concentrate here on situations in which local interaction structures and thus networks play a fundamental role. To our knowledge, there have been few experiments in which the population structure that has been recreated in the laboratory only allows for local interactions. Possibly among others, the works of My et al. [13]. of Keser et al. [14], of Berninghaus et al. [15], and of Cassar [12] are relevant in this context.

Keser at al. used a ring structure where each player has a neighbor on either side and a well-mixed structure for comparison. Their conclusions are that in the ring the preferred equilibrium is the risk-dominant one, while the payoff-dominant equilibrium was the more frequent result in the globally communicating population. This is in qualitative agreement with the theoretical predictions of Ellison [9] for the ring and of Kandori et al. [7] for the mixing case.

My et al. performed a comparative experimental study of Stag Hunt games with three different payoff matrices on mixing and structured populations. The population with local structure was composed by a circle of eight people where each player only interacted with her immediate right and left neighbors. They find that the first period modal choice of strategy, which is the payoff dominant one, plays a major role in the final outcome. In the global population case, the steady state generally lies in the same basin of attraction as the initial state. For the ring structure, the convergence to the risk-dominant outcome is more frequent than in the well-mixed case, especially when the payoff matrix values are such that the Pareto-superior basin shrinks. However, still often times the system converges to the Pareto-dominant state, which disagrees with the theoretical predictions of Ellison [9] based on noisy best reply dynamics. By examining the detailed history of play, the experimenters have found that, while in the global population subjects on average play myopic best response, in the ring with local structure a kind of imitation rule fits the data better than best reply.

In the study of Berninghaus et al. the authors find that a ring of eight or sixteen players leads to less coordination on the Pareto-efficient strategy 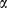 in the average than in groups of three completely connected players. In addition, with the same neighborhood size, grids of sixteen individuals are less effective in evolving coordination on the efficient equilibrium.

Our study is close to three other studies. The first study is the modeling and simulation work of Roca et al. [26], with some unavoidable limitations related to the small size achievable in the laboratory. Roca et al. studied cooperation and coordination on a couple of actual social networks and identified a different behavior: in one of the networks the Pareto-efficient strategy 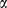 cannot propagate and the final equilibrium results in a dimorphic population. They attributed the phenomenon, which is deemed to be quite general, to the existence of *topological traps*, which are local network features characterized by local bridges [27] and scarcity of redundant paths. These structures make it difficult for any flow to easily propagate past the trap.

The second is the study of Cassar [12], which is the closest one from the standpoint of the present paper as it investigates network structures that are more realistic than the ring and the two-dimensional lattice, although the ring is also used in the experiments for comparison. Basically, the main finding of Cassar was that small-world networks were apparently the more conducive to coordination on the Pareto-efficient outcome, and she attributed this effect to the higher clustering of these networks with respect to random structures. We shall discuss her settings and results in more detail later.

The third article describes a recent experimental investigation on coordination games on various kinds of small-size networks [28]. The authors focus on equilibrium selection in these networks by the experimental subjects as compared with theoretical predictions. They found little support for the prediction that network effects have an influence on the emergence of a given equilibrium, as most groups coordinated on the efficient equilibrium irrespective of the network shape. However, they did find a difference between inexperienced subjects and those that have already played the game. After the first run, most groups coordinated on the efficient equilibrium very quickly. We also observed a strong initial bias towards playing strategy 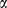 at the beginning of a run, a tendency that becomes stronger when subjects have gained some better understanding of the game (see section Results). Although the study is interesting, it is not really comparable to ours since the network size is very small (six) and this of course makes network effects more difficult to assess. Moreover, in most runs the participants had full information on the strategies and positions of all the other players, while in our case knowledge was effectively restricted to the first neighbors.

## Materials and Methods

### Ethics Statement

The use of human subjects in economics laboratory experiments has been approved by the ethics committee of the University of Lausanne. The participants were fully informed of the nature of the experiment and signed an informed consent to participate. Their anonymity has been guaranteed at all stages of the experiment.

### Network Design

**Figure 1:**
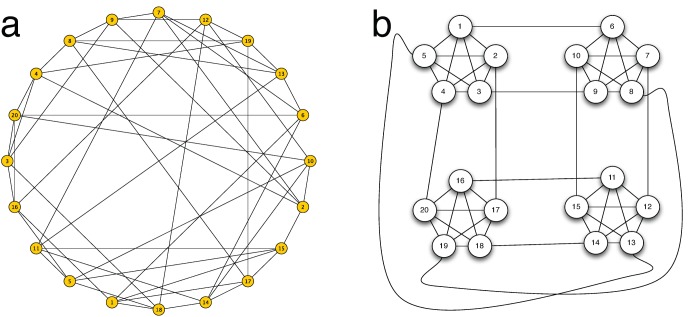
Network topologies used in the experiments. **a:** regular random graph; **b:** cliquish network.

We designed two basic network topologies containing 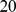 nodes each: a random network and a cliquish network. The random network, shown in Fig. 1a, is a regular random graph of degree five and is used as a baseline case in which no topological traps are present. The average clustering coefficient of this graph is 0.15. Two different matches of this topology, denoted by 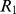 and 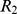 respectively, were tested with random relabeling of nodes between them. The idea behind a constant degree and relabeling of nodes is to avoid confusing the effect of the topology itself with that of the particular location of the node in it. This structure was used as a baseline against which the following is to be compared with respect to the game behavior.

The cliquish network is reproduced in Fig. 1b. Here each agent has the same number of neighbors (five) but she is more strongly connected to four of them, those belonging to the five-clique structure. This network was designed to reproduce the kind of topological traps of Roca et al. [26] introduced above, as the low number of links between cliques could, in principle, play the role of traps. This graph has an average clustering coefficient of 0.6, much higher than the random graph one (see above). Again, two matches of this topology were tested, that we denote as 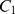 and 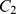.

In our opinion, the non-random network used in the experiments is much closer to actual social networks in its local structure than any previously used topology in the laboratory as rings and grids. Indeed, we found that comparable structures were independently used by Suri and Watts in a Web-based experimental study of public goods games [29].

### Specific Coordination Game

**Figure 2:**
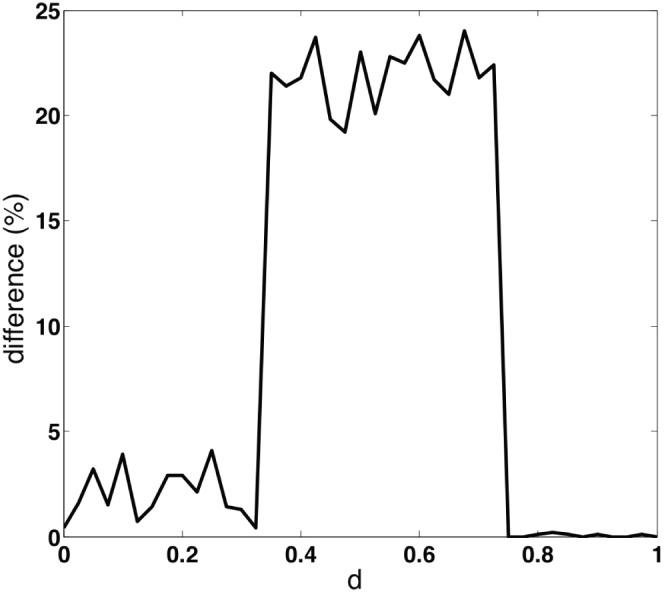
x-axis: payoff value 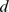; y-axis: absolute value of the difference between fractions of 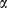 strategy at steady state in random and cliquish networks. The values are averages over 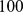 simulation runs for each network structure (see text).

With reference to Table 1, we have chosen a particular game in the coordination game space by fixing 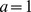, 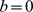, and 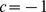. Then, using numerical simulations, we have varied the last parameter 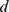 in the interval 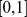 in such a way as to choose the value of 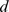 that approximately maximizes the difference in equilibrium fractions of 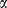 strategists and 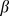 strategists between the random network and the cliquish one. Figure 2 shows the average results of 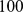 simulation runs on each of the networks, considering myopic best-response as update rule and an initial fraction of 0.7 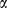-strategists. This last value is rather typical, being close to the initial frequencies of 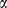 that have been observed in many laboratory experiments. Absolute values of the differences in steady state frequencies of 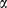 between the random graph and the cliquish network are reported on the y-axis as a function of 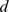 and are systematically higher for 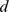 about 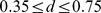. We fixed 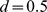, a value that lies in the interior of the interval that discriminates well between the cliquish and the random network. From these values, in order to obtain positive integer payoff values, we have performed an affine transformation of the matrix which leaves the NE invariant and leads to the matrix used in the experiment shown in Table 2.

**Table 2:** Specific coordination game used in the experiment.

	α	β
α	5, 5	1, 4
β	4, 1	3, 3

**Figure 3:**
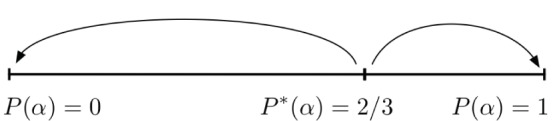
Basins of attraction for 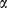 strategy (right basin) and for 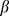 (left basin) when using the numerical payoffs of the experimental coordination game. 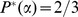
 is the unstable dimorphic equilibrium.

For the particular coordination game represented by this matrix, the mixed equilibrium is found to be 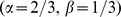 and the corresponding basins are sketched in Fig. 3. It is worth noting that the game used here is formally equivalent to the one employed in Cassar’s experiments [12].

We performed many numerical simulations on the graph structures shown in Fig. 1. We only present a summary of the results to save space; detailed data are available on request. Using pure best response as a strategy update rule on the cliquish network (Fig. 1b) and starting with 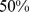 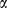-strategists the dynamics converges on all-*β* 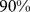 of the time, after 1000 repetitions; otherwise there is one clique of 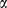 players that remain stable and the rest of the population plays 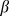. With an initial fraction of 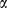 of about 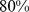, we never observe convergence on a 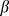 monomorphic population. Instead, in 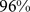 of the runs the game dynamics converged on dimorphic populations with cliques conquered either by 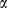 or 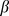 strategists and only 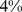 went to the all-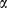 equilibrium. This last result shows that, in the absence of noise and errors of some kind, weak links do indeed cause freezing of the strategies in some parts of the network. In a series of runs we perturbed deterministic best response by adding a 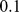 probability of making errors. In these conditions we always found convergence on the all-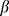 fixed point with both initial proportions. This is understandable as, with noise added, dimorphic configurations that were stable with pure best response, are destabilized and ultimately broken.

The baseline case of the regular random network of Fig. 1a is indeed rather different. With pure best response and an initial proportion of 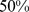 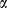, the dynamics always converges to all 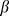. With 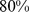 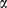 initially, convergence is on all 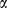 in 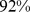 of the cases, with some runs converging to dimorphic populations. As soon as noise is added to pure best response, all the runs converged to the 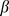 fixed point for both initial conditions.

### Implementation

We conducted a total of four experimental sessions that counted 20 participants each using the z-Tree environment [30]. Participants were recruited from a subject pool that includes students from several faculties. In each session, subjects played the coordination game in four different network topologies, and each topology lasted for 30 periods. In other words, the location of nodes in a network remained unchanged during 30 periods. Table 3 summarizes the order in which the different network topologies were implemented in each session.

**Table 3:** Summary of experimental sessions.

Session	Date	Subjects	Network 1	Network 2	Network 3	Network 4
1	03.10.2011	20	*C*_1_	*C*_2_	*R*_1_	*R*_2_
2	07.10.2011	20	*R*_1_	*R*_2_	*C*_1_	*C*_2_
3	07.10.2011	20	*C*_1_	*R*_1_	*C*_2_	*R*_2_
4	14.10.2011	20	*R*_1_	*C*_1_	*R*_2_	*C*_2_

Each period counted two stages. In the first stage, players had to select one of the two strategies, that we called “square” and “circle” instead of 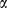 and 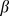 in order to avoid suggesting an implicit ranking. Subjects were allowed to take as much time as they wanted to reach a decision. The average response time was 2 seconds so most subjects were very fast in selecting their strategy. In the second stage, subjects observed on the screen their own choice, the number of neighbors that selected each strategy and their own gain of the period. In particular, they were never informed about their neighbors’ payoffs, nor about their individual strategy choices. This implies that payoff-based imitation rules are ruled out, since subjects cannot identify the most successful strategy.

Students read a detailed description of the experiment before the started playing the game. After reading the instructions, subjects had to respond to a set of control questions that insured common understanding of the game and the computation of payoffs. A translation of the instructions distributed to subjects is provided as supplementary material to this paper. After one round of 30 periods, subjects were informed that they would play the same game for another 30 periods, but that their neighbors (and their neighbors’ neighbors) would be different than the ones met in the previous round. They were not informed about the particular network topology of the society, but they were aware that they (and everyone else) would always play the game with five neighbors. Each session lasted for about 80 minutes and subjects earned, on average, 36.6 swiss francs, or about 30 EUR (37 USD).

## Results

### Aggregate Behavior

**Figure 4:**
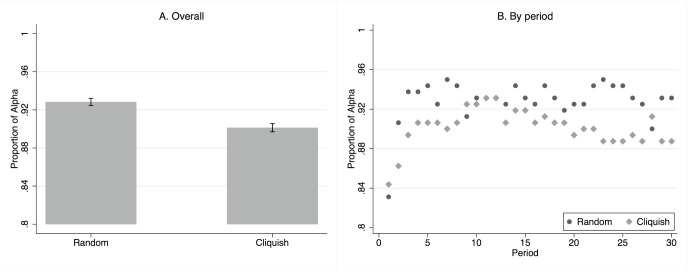
Proportion of 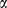-choices by network topology. Proportions are aggregated over all sessions and periods in A, and over session in B.

Fig. 4 reports the proportion of players choosing the efficient strategy 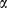 aggregated over all sessions and periods. Strategy 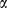 is the preferred choice at any time period in both topologies. Players on random networks coordinate a bit more often on the payoff-dominant strategy 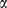 than players on the cliquish network (Fig. 4a). Yet, a standard t-test that accounts for clustering across individuals does not reject the hypothesis that the proportion of 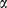-strategists is the same in both topologies.

When dis-aggregating by period (Fig. 4b), we see that subjects seem to understand payoff-dominance from the very first period, where no more than 20% of the population minimizes risk by choosing strategy 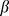. From this high initial rate of 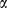-choices, convergence to almost full coordination on the payoff-dominant strategy is quite rapid.

### Estimating Individual Behavior

**Figure 5:**
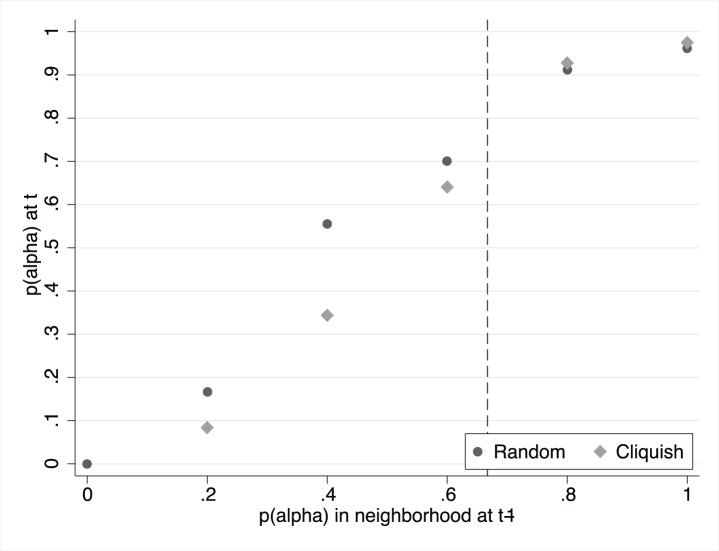
Proportion of 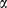-choices as a function of the fraction of neighbors that chose 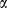 in the previous period. Dashed line at two thirds: at the left of the dashed line a myopic best replier chooses 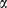 with probability zero; at the right of the dashed line a myopic best replier chooses 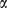 with probability one.

Our interest lies in understanding how subjects make their choices in response to their neighbor’s choices. The key graph compares the choice of strategy with the information received about the strategy choice of neighbors in the previous period. Fig. 5 plots the proportion of individual *α*-choices against the proportion of *α*-choices in the neighborhood in the previous period.

First of all, it is evident that most subjects are sensitive to the information regarding their neighbors’ choices, since their own decisions are correlated to it. Moreover, the effect of this information is monotone: the higher the proportion of *α*-choices in my neighborhood, the higher the probability that I also choose 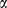 in the next period. Second, the effect seems to be non-linear or, more precisely, S-shaped. This would suggest that the biggest change in individual behavior occurs at intermediate values of the neighborhood’s distribution of *α*-choices. We now discuss how to use this evidence to infer whether individuals play myopic best-reply or not.

Remember that a player’s strategy 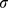 in any given period is a myopic best-reply if, assuming that the distribution of her neighbors’ strategies remains unchanged, it gives her a higher payoff than any other strategy.

Let 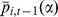 denote the fraction of neighbors of individual 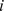 that chose 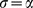 in the previous period. That is
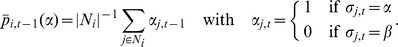
where 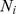 is the neighborhood of 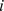 and 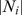 its cardinality (i.e. the degree of node 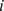). Given the payoffs used in this experiment, the (myopic) expected gains to subject 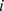 of choosing each strategy in period 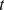 are



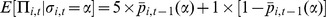


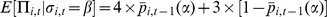



Thus, choosing the payoff-dominant strategy 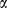 is a best response if.
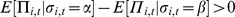


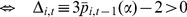
(1)


Borrowing the terminology used in Cassar [12], we will refer to the amount 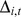 as the “payoff advantage” of choosing strategy *α*. Myopic best-reply means that an individual switches to playing 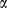 as soon as more than 2 out of 3 of her neighbors play *α*, i.e. as soon as the payoff advantage becomes positive.

If a subject uses myopic best-reply as update rule, we should observe 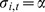 whenever (1) holds, i.e. the payoff advantage of choosing 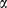 is positive, and 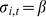 otherwise. Hence, we can estimate a model of the form
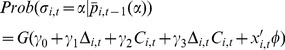
(2)


where 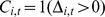, and 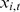 includes other relevant control variables. 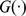 is a cumulative distribution function.

The parameter 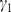 measures the effect of the payoff advantage on the probability of choosing 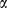, when this advantage is negative. If the subject follows myopic best-reply this parameter is zero because a negative payoff advantage, regardless of its size, should translate into a zero probability of choosing strategy 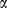. The parameter 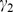 measures the discrete jump in the probability of playing 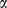 once more than two thirds of neighbors play 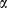. A myopic best-replier should go from choosing 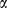 with zero probability to choosing it with certainty as soon as the payoff advantage becomes positive. This means that 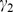 is 1 for a pure myopic best-replier. Finally, 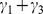 measures the effect of the payoff advantage on the probability of choosing 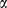, when this advantage is positive. Again, 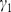 and 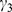 are zero for a player who plays myopic best-reply.

The specification (2) amounts to fitting the S-shaped relationship observed in the data of Fig. 5. To estimate the model, we consider 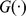 to be the uniform cdf and we include as control variables the lagged individual choice (to control for lock-in due to either inertia or unobserved heterogeneity), dummies for 5-period intervals and for the order in which the particular topology was played in the session. We have also explored estimating model (1) adopting a logistic specification for 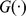, but did not pursue this approach since players ended up playing the 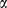 strategy with probability 1 in one repetition of the experiment. The logistic specification rules out such cases whereas the uniform specification allows for it. Table 4 reports the sensitivity of the probability of playing 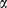 to the excess payoff (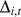), the change in that probability as the fraction playing 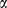 exceeds 2 out of 3 (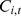), and the change in the sensitivity as the fraction of players playing 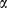 exceeds two thirds (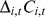). Inference is based on standard errors that account for clustering within individuals.

**Table 4:** Estimation of 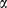-choice.

	Random	Cliquish
Δ*_i,t_*	0.202*	0.271***
	(0.085)	(0.046)
*C_i,t_*	0.065	0.088*
	(0.047)	(0.033)
Δ*_i,t_ C_i,t_*	−0.193*	−0.247***
	(0.084)	(0.046)
*α_i_*_,*t*–1_	0.572***	0.500***
	(0.097)	(0.099)
periods6to10	−0.023**	−0.008
	(0.007)	(0.009)
periods11to15	−0.022**	−0.009
	(0.008)	(0.007)
periods16to20	−0.023**	−0.010
	(0.008)	(0.008)
periods21to25	−0.014	−0.007
	(0.007)	(0.006)
periods26to30	−0.019	−0.008
	(0.010)	(0.008)
order2	0.029	0.006
	(0.018)	(0.009)
order3	0.018	0.003
	(0.011)	(0.008)
order4	0.022	0.002
	(0.014)	(0.009)
Session dummies	Yes	Yes
N	4640	4640

Notes: standard errors clustered by individual in parentheses.

*** p
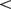
0.01,

** p
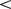
0.05,

* p
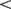
0.1.

Table presents marginal effects from a linear probability model. Δ*_i,t_* is the payoff advantage of choosing 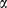 given the distribution of neighbors’ choices in previous period; *C_i,t_* = 1(Δ*_i,t_*>0); *α* = 1 (σ*_i,j_* = *α*); *C_i,t_* = 1(Δ*_i_ periods#* are dummies for 5-period intervals; *Order#* = 1 if network played in the #th order in a session; *cliquish* = 1 if cliquish topology, = 0 if random. Session dummies were included but turned out to be not significant.

The parameter 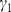 is estimated to be positive and significantly different from zero in both network topologies. This means that subjects are more likely to play 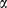 the less negative the payoff advantage of playing 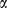 is. Second, there is some evidence of an increase in the probability of choosing 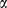 when the payoff advantage becomes positive, but the magnitude of the shift is small (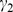 close to zero). Subjects are no longer sensitive to the payoff advantage as soon as more than two out of three of their neighbors have switched to playing *α*. These results reject the hypothesis that all subjects in the laboratory adopted pure myopic best reply as update rule.

Regarding the other variables, we see evidence of lock-in in that past individual choices are correlated to current ones. This could either be due to inertia or, as stressed by Berninghaus et al. [15], to unobserved heterogeneity. Players are significantly less likely to play 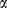 in rounds 6 to 20 (compared to rounds 1 to 5) in the random network. This effect is not present in the cliquish network. Moreover, the order in which the topologies were played do not matter.

Results suggest that players are somewhat more sensitive to their neighbor’s choices in cliquish networks than in random ones. Moreover, clustering could also be present across individuals within the same network. Table 5 reports an empirical specification that allows testing whether the updating rule differs between the cliquish and the random network. Column “All repetitions” reports results that use all repetitions in the experiment. Column “First repetition” reports results based on the first network topology that subjects played. All estimates report the standard errors clustered by individual in parentheses. The “first repetition” estimates report the standard error in brackets that allow for clustering at the individual level and for correlation between individual i’s choice in period t with the decisions of her neighbors in t−1. We do not report these standard errors in column 1 since we have been able to figured out how to calculate these standard errors only for the first repetition. The standard solution to account for clustering within sessions would be to assume arbitrary clustering within networks. While this assumption is realistic we do not have a sufficient number of sessions to apply this solution.

**Table 5:** Estimation of 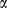-choice (interacted specification).

	All repetitions	First repetition
Delta	0.213	0.232
	(0.086)*	(0.087)**
	{0.011}***	{0.006}***
		[0.112]*
C	0.074	0.069
	(0.048)	(0.056)
	{0.016}*	{0.036}
		[0.062]
Delta*C	−0.197	−0.220
	(0.084)*	(0.090)*
		[0.105]*
Delta*cliquish	0.044	0.014
	(0.090)	(0.120)
		[0.118]
C*cliquish	0.003	−0.011
	(0.057)	(0.077)
		[0.081]
Delta*C*cliquish	−0.034	0.048
	(0.092)	(0.128)
		[0.112]
cliquish	−0.005	−0.015
	(0.057)	(0.072)
		[0.058]
l.alpha	0.540	0.482
	(0.092)***	(0.064)***
		[0.025]***
Constant	0.364	0.406
	(0.087)***	(0.073)***
		[0.023]***
N	9280	2320
r2	0.525	0.378
Order dummies	yes	no
Session dummies	yes	no
Period-interval dummies	yes	yes

Standard errors clustered by individual in parentheses. Restricted standard errors in square brackets (assuming independent sessions, no correlation between unconnected subjects in a session, no contemporaneous correlation between connected subjects, no correlation at lags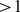 between connected subjects). * p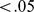, ** p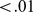, and *** p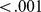 (t-distribution with 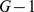 degrees of freedom, with 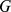 the number of clusters. The restricted standard errors consider the same distribution as the individual clustering.).

Table 5 shows two main results. First, the parameters in the update rule do not differ by topology. The terms 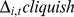, 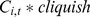, and 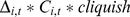 measure the difference in update rule parameters between the cliquish and the random network. None of these three parameters is significantly different from zero, regardless of the standard errors we use to perform the test. Second, column two indicates that our main result that subjects do not use myopic best reply is valid, again regardless of the type of standard error we use.

**Figure 6:**
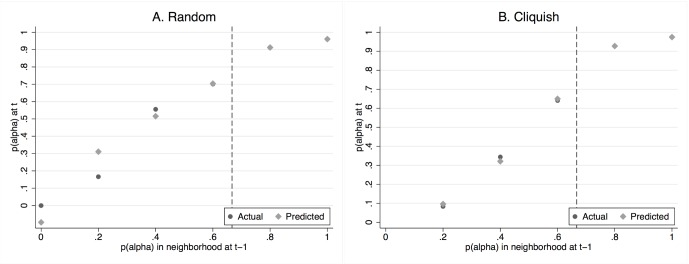
Actual and predicted proportion of 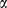-choices as a function of the fraction of neighbors that chose 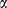 in the previous period.

Figure 6 plots the predicted probabilities of choosing 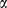 against the actual choices as a function of the previous period proportion of 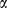-choices in the neighborhood. It appears that model (2) does a very good job in fitting the observed data, in particular for cliquish networks.

## Discussion

### Influence of the Network Structure

The analysis of the previous section has shown that human subjects do not use myopic best reply as an update rule. The analysis also shows that interaction structure has no influence on the aggregate fraction of individuals playing the efficient strategy *α*. We also do not find that network structure is important for how subjects adapt to their neighbor’s choices.

It is worthwhile to try to relate our observations with the theoretical predictions of Roca et al. [26]. In the experimental results we didn’t see clear signs of the fact that *topological traps* re-created in the laboratory by having cliques of players weakly connected to each other (see Fig. 1) do actually cause freezing of inefficient 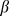 coordination zones or, equivalently, prevent 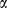 to spread further past the trap. There can be several reasons for this. First of all, while the strategy revision rule for the artificial agents was noiseless and always the same for all players, human subjects make errors and may try to experiment to gain more knowledge about the neighbors’ behavior. In particular, even a stable situation may become unstable if agents do not apply strict best response at the next time step. In fact, the update rule in Roca et al. [26] was payoff-based imitation dynamics, which cannot arise in any form here since the players are not informed about the payoffs of their neighbors. In addition, in the experiments strategy 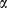 is always predominant in the first time step. Whatever the psychological or strategic reasons for that, it makes it more difficult for strategy 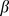 to gain a stronghold in a clique. Taken together, these two reasons make stable dimorphic states more difficult to attain than in theory or simulations based on simple and invariant protocol revision rules.

Moreover, while we were able to study small human networks of twenty people because of financial and equipment limitation, reasons that are common in this kind of laboratory work, simulations can be done on much larger systems in which the number of possible dimorphic stable states is higher. Finally, because of the size limitations of our networks, the degree correlation between agents at distance two and three, to which Roca et al. attribute an important role, are not meaningful. In spite of this, dimorphic situations in which some cliques were playing 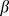 have been observed in the experiment. We identified such a state when at least one clique counted at least four *α*-strategists during the last five periods of a round and, at the same time, at least one clique counted at least four *β*-strategists during the last five periods of a round. Indeed, two out of the eight cliquish networks implemented ended up in a dimorphic stable state. In four of the cliquish networks all cliques became *α*-stable, while in the remaining two cliquish networks some cliques became *α*-stable while the others remained unstable. In terms of cliques, we find that 75 % of the cliques end up in a state where all players choose the 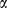 strategy in the final round, 12.5 % of the cliques have 4 out of 5 players choosing *α*, and the remaining cliques 12.5 % of the cliques were uniformly distributed between no-one choosing 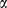 and 3 out of 5 choosing *α*.

**Figure 7:**
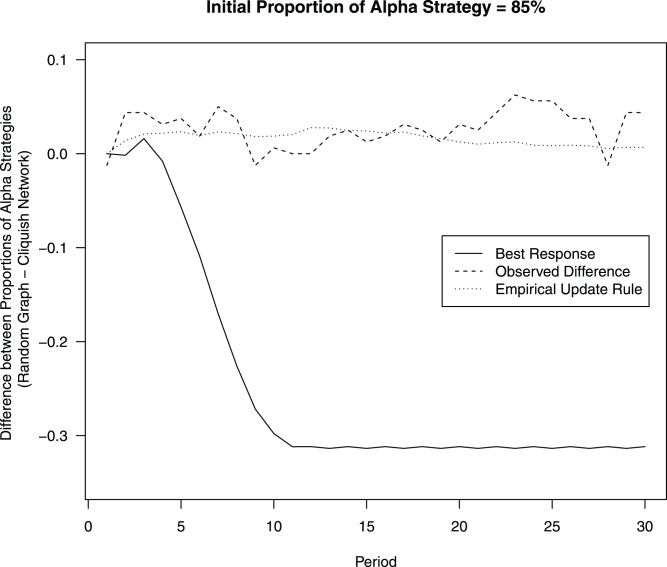
Difference in proportion of players choosing 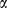 between random network and cliquish network. The figure reports simulations that use myopic best reply, the actual observed difference in the laboratory, and simulations that use the observed updating rule in Figure 5, averaged over topologies.

Interestingly, our results differ strongly from our own simulations that were based on the exact *same* network structures as in the laboratory and best response. The simulations predicted that from a 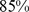 initial proportion of 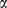-strategies, (i) most random networks end up in an all-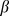 equilibrium while some of them end up in dimorphic states, and (ii) most cliquish networks end up in dimorphic states while a few of them end up in an all-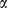 equilibrium. Figure 7 reports the difference in the proportion of players choosing 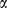 in the random network compared to the cliquish network assuming that the initial proportion of players choosing 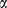 equals 85 % (this is the average fraction we observe in the laboratory over the four treatments). The simulations that assume myopic best reply as an update rule predict that the random network will have about 31 % fewer players choosing 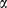 than the cliquish network from round 11 onwards. This prediction contrasts sharply with the main result from the laboratory experiment that the proportion of players choosing 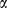 does not differ by topology. Interestingly, we are able to reproduce this laboratory result once we run simulations that implement the empirical updating rule that players use in the laboratory experiment (we implement the updating rule reported in Figure 5, averaged over topologies). This result highlights the usefulness of laboratory experiments to update the common behaviors implemented in the automata used in numerical simulations. See also the work of Grujic’ et al. [31] that does another step in this direction.

### Comparison with Related Experimental Results

Here we compare our results with the conclusions of a recent experimental study by Cassar [12]. Cassar’s work is the more related to ours since it is the only one we could find in the literature that explicitly tests for the influence of complex network structure on coordination games, with the exception of [28] which however deals with very small networks. She conducted a laboratory study in which eighteen subjects were virtually disposed as networks of three types: ring, small-world of the Watts-Strogatz class [32], and a random network, all with four neighbors per agent on average. The small-world networks in particular have a high clustering coefficient and a short path length. Aside from that, neither the ring nor the random or small-world networks featured cliques and weak links as in our settings (Fig. 1b). Each run consisted of eighty periods on average and three realizations of each network class were used. Ten runs were monitored in total for the three network types. The information available to the subjects was similar to ours including the number of randomly assigned neighbors and the fact that they were to stay the same during a given run. Cassar studied both the Prisoner’s Dilemma as well as the Stag Hunt games. Here we only comment about the Stag Hunt case. The payoff matrix for the coordination game in Cassar is equivalent to ours through an affine transformation. Most of Cassar’s runs started with high initial rates of 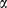 strategies and ended in the all 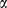 state, with the small-world networks being apparently the more conducive to coordination on the Pareto-efficient outcome. The differences, however, are small and their statistical significance is doubtful (see also the discussion of Cassar’s Prisoner Dilemma results in [29], where some doubt is cast on their statistical interpretation).

Our results also show a consistent preference for 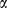 initially as well as later in the runs. However, we do not find that network structure plays a role. In the Prisoner’s Dilemma case, similar conclusions have been reached in a recent very large-scale experimental study on grids and scale-free networks by Gracia-Lázaro et al. [33] in which the authors conclude that the level of cooperation reached in both structures is the same. Likewise, Suri and Watts conclude that the network topology had no statistically significant effect on the average contribution in a public goods game [29].

Cassar also analyzed the individual player decision making and came to the conclusion that best reply and inertia are significant in explaining behavior. While we also find evidence of inertia, we reject that subjects in our experiment use best-reply as update rule.

### Conclusions

We study the role of network topology for coordination decisions in a Stag Hunt game. Numerical simulations of the setting suggest that populations of 20 players will end up in a dimorphic state more often in the cliquish network than in the random network. Also, players choose the efficient strategy 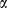 more often in the random network. While we find that human subjects in a laboratory setting do converge more often to dimorphic states in cliquish networks than in random networks, there is no difference in terms of the proportion coordinating on the efficient outcome between the two topologies. Moreover, subjects do not use best-reply as update rule. Numerical simulations agree with laboratory results once we implement the actual updating rule that human subjects in our laboratory experiments use.

This evidence suggests that numerical simulations can be a useful tool to understanding coordination in small scale societies. However, they should incorporate more empirical knowledge on their strategy update functions, which are currently too simplistic. These methods can then be updated and improved, hopefully not only for small scale but also for large scale societies – settings where laboratory studies are hard and very costly to implement.
